# Effects of Molybdenum Supplementation in the Form of Ammonium and Sodium Salts on Trophoblast Cell Physiology and Gene Expression In Vitro

**DOI:** 10.3390/jdb13010008

**Published:** 2025-03-05

**Authors:** Vladimira Foteva, Joshua J. Fisher, Yixue Qiao, Roger Smith

**Affiliations:** 1 Mothers and Babies Research Program, Hunter Medical Research Institute, Newcastle, NSW 2305, Australia; 2 School of Medicine and Public Health, University of Newcastle, Newcastle, NSW 2308, Australia; 3 Wisdom Lake Academy of Pharmacy, Xi’an Jiao Tong Liverpool University, Suzhou 215123, China

**Keywords:** micronutrients, molybdenum, placenta, HTR8/SVneo cells

## Abstract

Molybdenum is an essential trace element sourced during pregnancy from the maternal diet. Studies regarding molybdenum have primarily focused on overexposure in animal and cell culture studies. The effects of molybdenum supplementation on placental function are unknown. An immortalised trophoblast cell line was used to examine the placental cellular response to molybdenum in its bioavailable form as molybdate. Cells of the extravillous trophoblast first-trimester cell line HTR8-SVneo were cultured in complete cell media in the presence of 10 nM to 1 mM of ammonium molybdate or sodium molybdate. Following the addition of the molybdate salts, cell growth, viability, and several gene pathways were monitored. Sodium molybdate salt in doses from 10 nM to 1 mM did not affect cell growth or viability. Exposure to ammonium molybdate at a 1 mM concentration significantly decreased cell growth and viability (*p* < 0.05). Gene pathways involving molybdoenzyme expression, molybdenum cofactor synthesis, antioxidant response, and angiogenesis were affected following supplementation, although these effects differed depending on the dose and molybdate salt utilised. Molybdoenzyme activity was not affected by supplementation in a dose-dependent manner. The results indicate sodium molybdate is a more appropriate salt to use in vitro, as ammonium molybdate exposure reduced cell viability and growth and downregulated the expression of antioxidant genes *NFE2L2* (*p* < 0.01), *SOD1* (*p* < 0.001) and *SOD2* (*p* < 0.001), suggestive of an inflammatory response. Sodium molybdate affected gene, protein, and activity levels of molybdoenzyme, antioxidant, and angiogenic molecules in vitro. This work demonstrates that sodium molybdate supplementation has pleiotropic effects in vitro and is well tolerated by placental cells at a range of nanomolar and micromolar concentrations.

## 1. Introduction

Molybdenum is an essential micronutrient in the maternal diet, ubiquitous in the environment and found in a variety of foods [1,2]. Molybdenum, in the form of its soluble oxyanion molybdate ion [MoO_4_]^2−^, is biologically active and forms the active site of several enzymes with diverse physiological functions, termed molybdoenzymes [3,4]. The five mammalian molybdoenzymes discovered to date are aldehyde oxidase, xanthine oxidase, sulphite oxidase, and mitochondrial amidoxime reducing component 1 and 2, which utilise molybdenum within a pterin-based scaffold known as the molybdenum cofactor (Moco) [5]. Molybdoenzymes have roles in the catabolism of aldehydes, purines, sulphur-containing amino acids, and amidoximes [1,6]. However, research regarding molybdenum transport and uptake in mammalian cells remains limited; a molybdate transporter protein family termed Molybdenum Transporter 2 (MoT2) has been detected in humans, with additional molybdate transporters speculated (reviewed extensively in [1]), although they have not been linked to gestation or placental development in the literature. Related to pregnancy, molybdenum has been detected analytically using X-ray fluorescence microscopy, mass spectrometry, and atomic absorption spectrophotometry in placental tissue, umbilical cord tissue, maternal serum, and amniotic fluid [7,8,9,10].

Despite the essential nature of the element and its associated enzymes, the literature concerning molybdenum supplementation throughout gestation is scarce and contradictory [1,2,3,4]. In population-wide studies, molybdenum levels in the maternal circulation have been both positively and negatively associated with gestational diabetes, neural tube defects, and oxidative stress during pregnancy [1]. Molybdoenzyme expression and activity in the form of aldehyde oxidase and xanthine oxidase is associated with increased oxidative stress and placental ageing [11,12], while a deficiency of the molybdenum cofactor results in neurodegeneration and lethality for the organism due to the accumulation of toxic sulphites [13]. Elucidating the precise nature of molybdenum’s effects on cell systems is complicated by its trace element status. This renders molybdenum difficult to study in a cost-effective manner in large population groups, and this is further exacerbated by the lack of existing standardised clinical tests or biomarkers to monitor molybdenum insufficiency, or excess, although biomarkers for isolated sulphite oxidase deficiency and molybdenum cofactor deficiency exist [14].

To date, the majority of the literature remains focused on overexposure, with supraphysiological exposure several orders of magnitude above physiological intake reviewed elsewhere [15]. Briefly, studies of sodium and ammonium molybdenum salt exposure in the range of 100 mg/kg or high millimolar (mM) doses were found to induce apoptosis and chromosomal damage and inhibit proliferation in rodent studies and mammalian cells, respectively [16,17]. Molybdenum, in the form of the inorganic soluble salt sodium molybdate, acts as an antioxidant and cytoprotectant in vitro and in vivo in mouse and rat cells, at dosages ranging from 5 mg/L to 100 mg/kg/day [16,18,19]. In contrast, the daily intake of molybdenum is estimated to be ~80–200 μg/day in human adults, and molybdenum concentrations in biological matrices range between 0.5–10 µM (tissue) and 10–500 nM (serum), indicating that overall molybdenum stores are variable dependant on tissue [1,20]. There are limited data available on the relationship between molybdenum and cell physiology at low but physiologically relevant concentrations in the nanomolar and micromolar ranges described above, and within the reproductive sphere these studies have primarily focused on oocyte function and spermatogenesis [16,19]; to our knowledge, there are no studies examining the effects of molybdenum exposure at physiological doses in placental cells. The placenta acts as the maternal–foetal interface, mediating nutrient availability, hormone production, and waste excretion, and is integral to the health and development of the foetus [21]. Considering the versatile chemistry and physiological roles of molybdenum in cell systems, this element may play integral and multifaceted roles in placental physiology and function and in the aetiology of placental-mediated pregnancy pathologies such as foetal growth restriction and pre-eclampsia.

In order to investigate the actions of this essential micronutrient during gestation, the HTR-8/SVneo cell line was used as an in vitro model of placental extravillous trophoblast cells. The objective of this study was to investigate the effects of soluble, commercially available molybdate salts on pathways involved in the function and development of a placental cell line, at the gene, protein, and activity levels, and specifically determine the effects on molybdoenzyme activity in extravillous trophoblast cells.

## 2. Materials and Methods

### 2.1. Cell Culture

The human trophoblast HTR-8/SVneo cell line was obtained from the American Type Culture Collection (ATCC), Manassas, VA, USA. Cells were cultured in Roswell Park Memorial Institute (RPMI) 1640 media (ThermoFisher Scientific, Waltham, MA, USA) supplemented with 10% foetal bovine serum (Sigma Life Sciences, Australia), 1% Antimycotic-Antibiotic solution (Sigma-Aldrich, St. Louis, MO, USA), 100 mM sodium pyruvate (Gibco, ThermoFisher Scientific, New York, NY, USA) and 200 mM L-Glutamine (Gibco, ThermoFisher Scientific, New York, NY, USA) in a humidified incubator containing 5% CO_2,_ at 37 °C. Cells in the logarithmic growth phase were seeded in sterile tissue culture plates and flasks for subsequent experimentation. Molybdate salts sodium molybdate dihydrate, Na_2_MoO_4_·2H_2_O, (Sigma-Aldrich) and ammonium molybdate tetrahydrate, (NH_4_)_6_Mo_7_O_24_·4H_2_O, (Sigma-Aldrich) were dissolved in MilliQ water and syringe-filtered (0.2 µM pore) before their addition to cell culture. For the investigation of enzymatic activity, gene expression, and protein expression, cells were seeded in 75 cc flasks at a concentration of 2.1 million cells per flask and treated with salts at 24 h, and the cells collected at 48 h using cell scrapers, in PBS (24 h incubation in molybdenum supplementation). Cells were resuspended in TRIzol reagent (Ambion by Life Technologies, ThermoFisher Scientific, California, CA, USA) for RNA extraction [22] and resuspended in lysis buffer consisting of 1% Triton X-100, 0.01% Brij-35, 1X protease inhibitor (Roche, Sigma-Aldrich, Mannheim, Germany), and 1X phosphatase inhibitor (Roche, Sigma-Aldrich, Mannheim, Germany) in PBS for Western blot analysis. Cells were resuspended in enzyme lysis buffer consisting of 50 mM Tris Hydrochloride (Invitrogen), 1 mM M EDTA (Invitrogen), and 0.5% glycerol (UNIVAR, Sigma-Aldrich) in MilliQ water for enzymatic analysis.

### 2.2. Cell Viability Assay

Cell viability was determined following treatment with molybdate salts, via an MTT, 3-(4,5-dimethylthiazol-2-yl)-2,5-diphenyltetrazolium bromide assay as previously described [23,24]. HTR-8/SVneo cells were cultured in complete media in 96-well culture plates for 24 h at 2 × 10^4^ cells/well. Then, the cells were incubated in complete media supplemented with molybdate salts at concentrations ranging between 10 nM and 1 mM for a subsequent 24 h. The cells were then washed with PBS and incubated with fresh media and 50 μL of 1 mg/mL MTT (Invitrogen) at 5% CO_2_ and 37 °C for 3.5 h. The final MTT concentration per well was 0.25 mg/mL. Subsequently, the media were replaced with 125 µL of DMSO per well. The optical density values were acquired with a spectrophotometer microplate reader (SpectroStarNano, BMG Labtech, AUS) at 560 nm.

### 2.3. Cell Growth Curves

HTR-8/SVneo cells were seeded in 96-well culture plates at 2 × 10^4^ cells per well in complete media and allowed to adhere for 4 h before the addition of molybdenum salts. Following treatment, the cells were incubated in an Incucyte^®^ Zoom Heracell240i Incubator (ThermoFisher Scientific) for 36 h. Images were taken every 3 h, with 4 images per well and 2 wells per experimental repeat, and curves were calculated of the percentage confluence using phase contrast via Incucyte Zoom software. All data points were normalised to confluence at 0 h as previously described [25].

### 2.4. Quantitative PCR

Total RNA was isolated from treated and control cell flasks with TRIzol^TM^ reagent and column-purified using the Direct-zol^TM^ RNA MiniPrep kit (Zymo Research, Irvine, CA, USA). The isolated RNA was treated with DNase I (Qiagen, Hilden, Germany) to remove genomic contamination before use. RNA quality and concentration were assessed utilising an ND-1000 spectrophotometer and A_260_/A_280_ and A_260_/A_230_ ratios. Five hundred nanograms of cDNA was synthesised per sample with the SuperScript^TM^ IV First-Strand Synthesis System (Thermo Fisher Scientific, Australia) according to the manufacturers’ instructions, using Oligod (T) primers. A real-time PCR analysis was performed using QuantStudio 6 and 7 Real-Time PCR Systems (Quant Studio 6/7 Flex Real-Time PCR Systems, Applied Biosystems, AUS, Waltham, MA, USA) and SYBR™ Green PCR Master Mix (Applied Biosystems, Waltham, MA, USA). Each reaction contained 6 µL of SYBR Green PCR master mix (Life Technologies, Carlsbad, CA, USA), primers, 1 ng cDNA, and water to 10 µL. The primers were synthesised by KiCqStart™ (Sigma Aldrich, St. Louis, MO, USA). The primer sequences used in this study are shown in Table 1. All samples were measured in duplicate, and the data were analysed using the 2^−△△Ct^ method. The geomean of housekeeper genes beta-actin and GAPDH was used to calculate the relative abundance of mRNA for genes of interest.

### 2.5. Western Blotting

Quantification of the protein content from the cell extracts was performed using a BCA Protein Assay kit (Pierce, Rockford, IL, USA) following the manufacturers protocol. Bovine serum albumin (BSA) was used as the protein standard for the construction of a standard curve, from which unknown protein concentrations could be extrapolated. Protein concentrations are expressed as mg of protein per mL of cell extract (mg/mL).

Lysed cells in buffer were centrifuged at 14,000× *g* for 5 min at 4 °C and supernatant collected for analysis. Protein extract was treated with 1X Reducing Agent (ThermoFisher Scientific) and 1X LDS Buffer (ThermoFisher Scientific) and heated at 70 °C for 10 min. Protein extract (10 μg per well) was separated by electrophoresis in NuPAGE 4–12% Bis-Tris precast 12-well gels (NuPAGE, ThermoFisher Scientific) for 60 min at a constant 200 V (Bio-Rad Power Pac 300, California, CA, USA). Resolved proteins were transferred onto nitrocellulose membranes using the iBlot 2 Western Blot Dry Transfer System (Invitrogen, AUS, Waltham, MA, USA). Total protein was stained using Ponceau S (0.1% Ponceau, 5% Acetic Acid) and imaged using an Amersham Imager 600 (GE Healthcare). All incubations were performed on a rocking platform during immunoblotting. Membranes were blocked for 1 h at room temperature with 1% BSA in tris-buffered saline with 0.1% Tween-20 (TBST). Membranes were then incubated overnight at 4 °C with primary antibody in 1% BSA TBST. The antibodies used were for aldehyde oxidase AOX1 (1:1000, Protein Tech), sulphite oxidase SUOX (1:1000, Abcam, Cambridge, UK), xanthine dehydrogenase XDH (1:500, Abcam), and beta-actin, ACTB (1:2000, Abcam). The membranes were then washed three times with TBST and incubated at room temperature with anti-rabbit secondary antibody for all except the beta-actin, which was exposed to anti-mouse in 1% BSA in TBST for 90 min (1:2000, Cell Signalling). After 3 further washes with TBST, the immunoreactive bands were developed in Luminata reagent (Merck Millipore, Billerica, MA, USA) and detected via an Amersham Imager 600. A densitometric quantification of the immunoreactive bands was also performed using Amersham Imager 600 analysis software (GE Healthcare). The abundance data (optical density, arbitrary units) for each protein of interest were normalised to β-actin.

### 2.6. Enzymatic Assays

#### 2.6.1. SOD Assay

Superoxide dismutase activity was quantified using Cayman Chemical’s SOD Assay kit (Sapphire Bioscience, Redfern, Australia) in 96-well culture plates according to the manufacturer’s instructions as previously published [26]. Briefly, the kit utilises a tetrazolium salt for the detection of superoxide radicals generated by xanthine oxidase and hypoxanthine. SOD activity was calculated using a standard curve. Absorbance was read at 450 nm on the SpectroStar Nano plate reader. Samples were diluted 1:4 in original storage buffer. Superoxide dismutase activity was normalised to protein concentration.

#### 2.6.2. Catalase Assay

Catalase activity was quantified using Cayman Chemical’s CAT Assay kit (Sapphire Bioscience, Redfern, Australia) in 96-well culture plates according to the manufacturer’s instructions. Briefly, in this assay the catalase enzyme reacts with methanol in the presence of hydrogen peroxide, producing formaldehyde, which is measured colorimetrically with Purpald^TM^ as the chromogen. Absorbance was read at 540 nm on the SpectroStar Nano plate reader. Catalase activity was normalised to protein concentration.

#### 2.6.3. Xanthine Oxidase Assay

Xanthine oxidase activity was quantified using Cayman’s XO Fluorometric Assay kit in 96-well culture plates according to the manufacturer’s instructions. Briefly, xanthine oxidase produces hydrogen peroxide during the oxidation of hypoxanthine, which reacts with ADHP (10-acetyl-3,7-dihydroxyphenoxazine) in the presence of horseradish peroxidase to produce the fluorescent compound resorufin. Fluorescent activity was read on the FluoroStar Nano plate reader at an excitation wavelength of 544 nm and emission wavelength of 590 nm. Xanthine oxidase activity was normalised to protein concentration.

### 2.7. Statistical Analysis

All the data are presented as mean ± SEM. The Shapiro–Wilk test and Kolmogorov–Smirnov test were used to estimate the normality distribution of the data. The outlier tests Grubbs and ROUT were performed for each data set. Statistical differences were determined by one-way ANOVA followed by Dunnett’s post hoc test (Graph Pad Software Inc. San Diego, CA, USA). Differences were considered significant at *p* < 0.05.

## 3. Results

### 3.1. Effects of Molybdate Salts on HTR-8/SVneo Cell Viability

**Figure 1:**
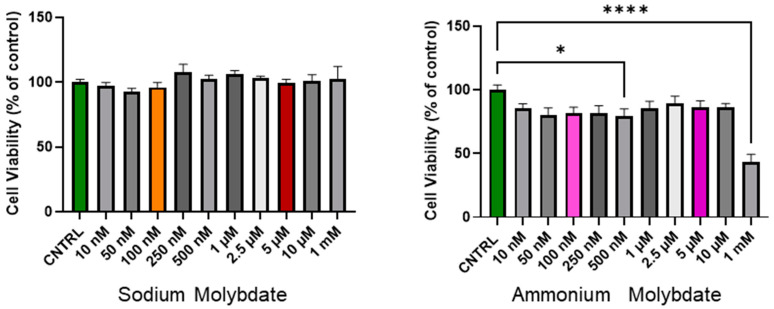
Cell viability of HTR-8/SVneo cells following incubation with sodium molybdate and ammonium molybdate at varying concentrations for 24 h. MTT performed at 48 h post seeding. Data presented as mean + SEM. N = 4 (sodium molybdate), 3 (ammonium molybdate) experiments performed in technical quadruplicate. A significant *p* value less than 0.05 is signified by an asterisk (*). **** signifies *p* < 0.0001. Molybdenum salt concentrations of 100 nM and 5 uM are highlighted in colour (orange, red for sodium molybdate; pink, purple for ammonium molybdate) as selected concentrations for subsequent experiments.

The cell viability following 24 h incubation with increasing concentrations of sodium and ammonium molybdate (at nanomolar and micromolar ranges) is shown in Figure 1. Sodium molybdate did not significantly affect cell viability in the nanomolar, micromolar, or millimolar dosage range.

In contrast, ammonium molybdate significantly reduced cell viability at a 500 nM supplementation dose (*p* < 0.05), and reduced cell viability to below 50% at 1 mM supplementation (*p* < 0.0001). The micromolar supplementation of ammonium molybdate did not alter cell viability significantly, though a non-significant decrease was noted with exposure across all doses.

### 3.2. Effects of Molybdate Salts on Cell Proliferation

**Figure 2:**
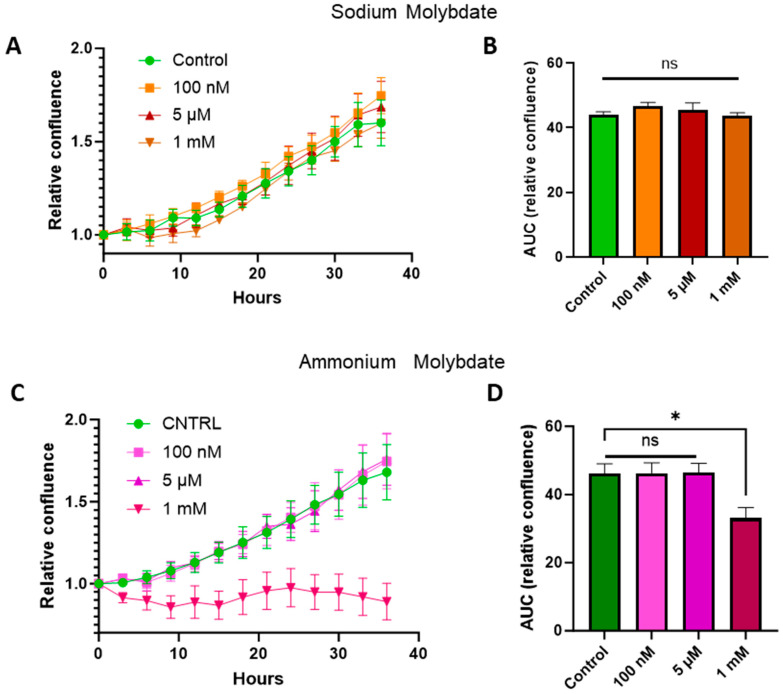
Proliferation of HTR-8/SVneo cells following supplementation with sodium molybdate (**A**) and ammonium molybdate (**C**) at three different concentrations over 36 h. Images were captured every 3 h, and percentage confluence was calculated for each well. Area under curve analysis performed for sodium molybdate (**B**) and ammonium molybdate (**D**) proliferation data. All data points are normalised to starting confluence (0 h). Green represents control data, orange and red represent sodium molybdate data, and pink and purple represent ammonium molybdate data. Significant *p* value less than 0.05 is signified by asterisk (*), ns indicates not significant. Data presented as mean + SEM. N = 4 from 2 independent experiments performed in duplicate.

Treatment with sodium molybdate salts did not significantly alter cell viability; therefore concentrations from the nanomolar (100 nM) and micromolar range (5 μM) were selected, alongside a supraphysiological dose (1 mM), for subsequent cell growth curve experiments for both ammonium molybdate and sodium molybdate salt applications. The proliferation of HTR-8/SVneo cells following molybdate salt supplementation was monitored via IncuCyte. Cells were seeded and allowed to adhere, and cell confluency, as a proxy measurement of proliferation, was monitored for 36 h. An area under curves analysis showed that the proliferation of HTR-8SV/neo cells was unaffected at both low and high dosages of sodium molybdate (Figure 2A,B) and ammonium molybdate (Figure 2C,D) but was significantly reduced (*p* = 0.0214) at a supraphysiological ammonium molybdate concentration in line with viability assessments (Figure 1). Cell confluence remained static at this concentration.

### 3.3. Effects of Molybdate Salts on Molybdenum Containing Enzyme Gene Expression, Protein Levels, and Enzyme Activity

**Figure 3:**
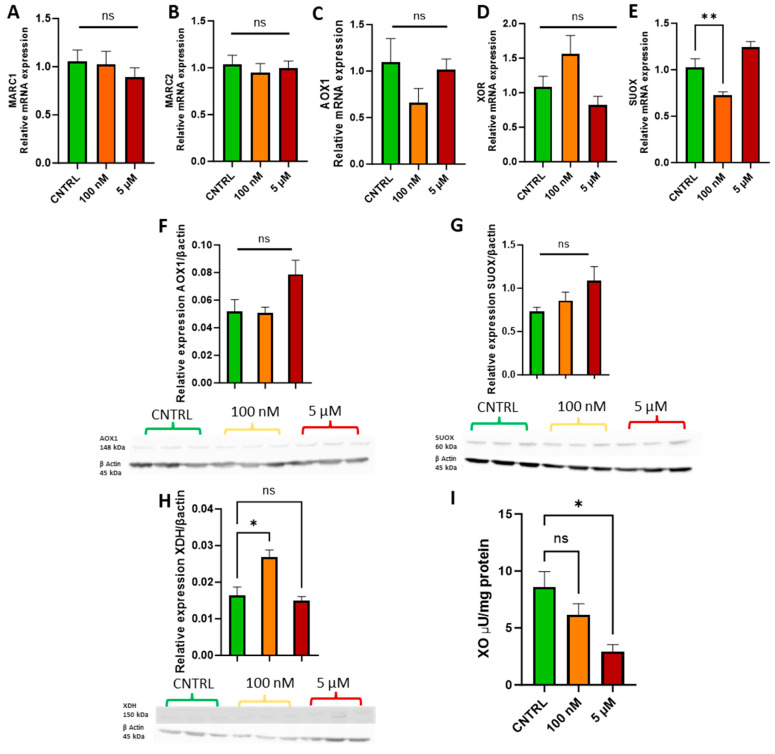
Effects of sodium molybdate salt supplementation on molybdoenzyme gene expression, protein expression, and activity. The effects of sodium molybdate supplementation on (**A**) MARC1, (**B**) MARC2, (**C**) AOX1, (**D**) XOR, and (**E**) SUOX expression following incubation for 24 h are presented. The effects of sodium molybdate supplementation on relative protein levels of (**F**) AOX1, (**G**) SUOX, and (**H**) XDH are presented, normalised to housekeeping protein beta-actin (shown). Xanthine oxidase activity (**I**) was also investigated under the same experimental parameters and normalised to mg protein. A significant *p* value less than 0.05 is signified by an asterisk (*); ** signifies *p* < 0.01, ns indicates not significant. Data presented as mean + SEM. N = 5–8 separate experiments for gene expression analyses. N = 3 separate experiments for enzymatic activity and protein content analyses.

Following the cell growth curve analysis, physiologically relevant doses of 100 nM and 5 µM were selected to assess whether sodium molybdate supplementation affects molybdoenzyme gene expression (Figure 3A–E), protein levels (Figure 3F–H), and activity in trophoblast cells (Figure 3I).

Supplementation with 100 nM and 5 µM sodium molybdate salt did not significantly affect the gene expression of mitochondrial amidoxime reducing components 1 and 2, *MARC1/2* (Figure 3A,B), or the cytosolic enzymes aldehyde oxidase, *AOX1* (Figure 3C), and xanthine oxidoreductase, *XOR* (Figure 3D). A supplementation of 100 nM significantly decreased the expression of mitochondrial enzyme sulphite oxidase, *SUOX* (*p* = 0.007) (Figure 3E), but did not significantly affect expression levels at the increased concentration of 5 µM. Protein levels of aldehyde oxidase (Figure 3F) and sulphite oxidase (Figure 3G) were not affected by either dosage of sodium molybdate. Xanthine oxidoreductase expression was, however, noted to increase non-significantly at the mRNA level following 100 nM sodium molybdate supplementation, which was reflected in significantly increased xanthine dehydrogenase (a precursor to xanthine oxidase) protein expression (*p* = 0.0125) (Figure 3H) at the same dose, although this was not conferred at the increased concentration of 5 µM. In contrast, xanthine oxidase activity decreased in molybdate supplemented trophoblast cells; the baseline activity of xanthine oxidase was 8.59 ± 1.92 μU/mg, which decreased to 6.14 ± 1.39 μU/mg at 100 nM sodium molybdate supplementation and significantly (*p* = 0.0144) decreased to 2.94 ± 0.84 μU/mg at 5 µM sodium molybdate supplementation.

**Figure 4:**
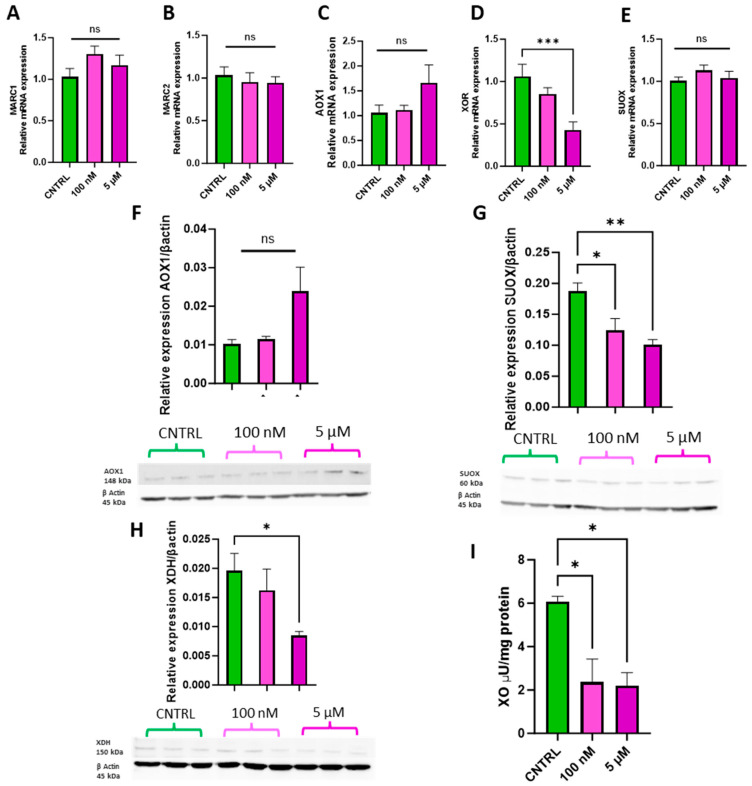
Effects of ammonium molybdate supplementation on molybdoenzyme gene expression, protein expression, and activity. The effects of ammonium molybdate supplementation on (**A**) MARC1, (**B**) MARC2, (**C**) AOX1, (**D**) XOR, and (**E**) SUOX gene expression following incubation for 24 h are presented. The effects of ammonium molybdate supplementation on relative protein levels of (**F**) AOX1, (**G**) SUOX, and (**H**) XDH are presented, normalised to housekeeping protein β-actin (shown). Xanthine oxidase activity (**I**) was also investigated under the same experimental parameters and normalised to mg protein. A significant *p* value less than 0.05 is signified by an asterisk (*); ** signifies *p* < 0.01, *** signifies *p* < 0.001, ns indicates not significant. Data presented as mean + SEM. N = 5–8 separate experiments for gene expression analyses. N = 2–3 separate experiments for enzymatic activity. N = 3 separate experiments for protein content analyses.

The physiologically relevant doses of 100 nM and 5 µM were selected to assess whether ammonium molybdate supplementation affects molybdoenzyme gene expression (Figure 4A–E), protein levels (Figure 4F–H), and activity in trophoblast cells (Figure 4I).

Supplementation with 100 nM and 5 µM ammonium molybdate salt did not significantly affect the gene expression of *MARC1*, (Figure 4A) *MARC2* (Figure 4B), *AOX1* (Figure 4C), or *SUOX* (Figure 4E). A supplementation of 5 µM ammonium molybdate significantly decreased the expression of xanthine oxidoreductase, *XOR* (*p* = 0.0008) (Figure 4D). Protein levels of aldehyde oxidase (Figure 4F) paralleled the gene expression analyses and were not affected by either dosage of ammonium molybdate. Sulphite oxidase protein levels, however, were significantly reduced following both 100 nM (*p* = 0.036) and 5 µM (*p* = 0.0099) supplementation. Xanthine dehydrogenase expression was likewise significantly reduced by the higher concentration of ammonium molybdate at 5 µM (*p* = 0.0476), but not by 100 nM supplementation, reflecting gene expression changes. Xanthine oxidase activity also decreased in ammonium molybdate-supplemented trophoblast cells; the baseline activity of xanthine oxidase was 6.08 ± 0.346 μU/mg, which significantly decreased to 2.37 ± 1.06 μU/mg with 100 nM ammonium molybdate supplementation and 2.21 ± 0.59 μU/mg with 5 µM ammonium molybdate supplementation (*p* = 0.0194, *p* = 0.0169 respectively).

### 3.4. Effects of Molybdate Salts on Molybdenum Cofactor Synthesis Gene Expression

**Figure 5:**
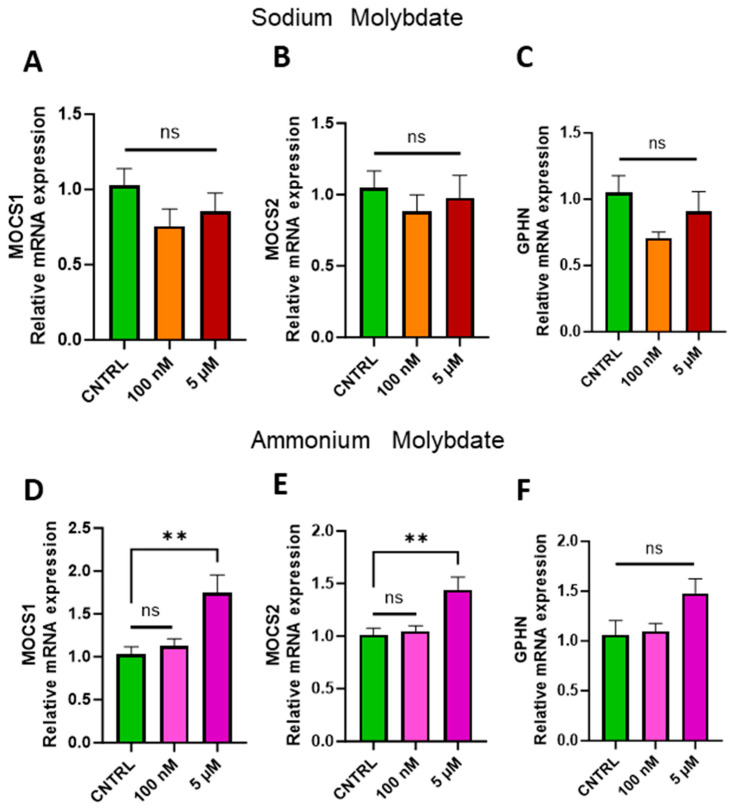
Effects of molybdate salt supplementation on molybdenum cofactor gene expression. The effects of sodium molybdate supplementation on (**A**) MOCS1, (**B**) MOCS2, and (**C**) GPHN gene expression and ammonium molybdate supplementation on (**D**) MOCS1, (**E**) MOCS2, and (**F**) GPHN expression following incubation for 24 h are presented. ** signifies *p* < 0.01, ns indicates not significant. Data presented as mean + SEM. N = 7–8 separate experiments for gene expression analyses.

Molybdenum must be complexed within a pterin-based scaffold at the active site of each enzyme in order to be catalytically active, together forming the molybdenum cofactor (Moco). This scaffold, termed molybdopterin (MPT), requires the expression of three genes: Molybdenum Cofactor 1, *MOCS1*; Molybdenum Cofactor 2, *MOCS2*, and Gephyrin, *GPHN* [3]. Mutations to any one of these genes result in molybdenum cofactor deficiency and lethality for the organism [4]; in order to assess the cell’s uptake and storage response to molybdenum supplementation in a non-stressed in vitro environment, we examined the expression of these three genes. No statistically significant difference was found in the expression of these molybdenum cofactor genes following supplementation with sodium molybdate at 100 nM or 5 µM concentrations (Figure 5A–C). However, the expression of *MOCS1* and *MOCS2* (Figure 5D,E), while unaffected following 100 nM supplementation, were both significantly increased with exposure to the higher dosages of 5 µM ammonium molybdate (*p* = 0.0023, *p* = 0.0033, respectively). The expression of GPHN, while not significantly affected by either dosage of ammonium molybdate, was likewise upregulated in the 5 µM supplemented group (Figure 5F).

### 3.5. Effects of Molybdate Salts on Antioxidant Gene Expression and Antioxidant Enzyme Activity

**Figure 6:**
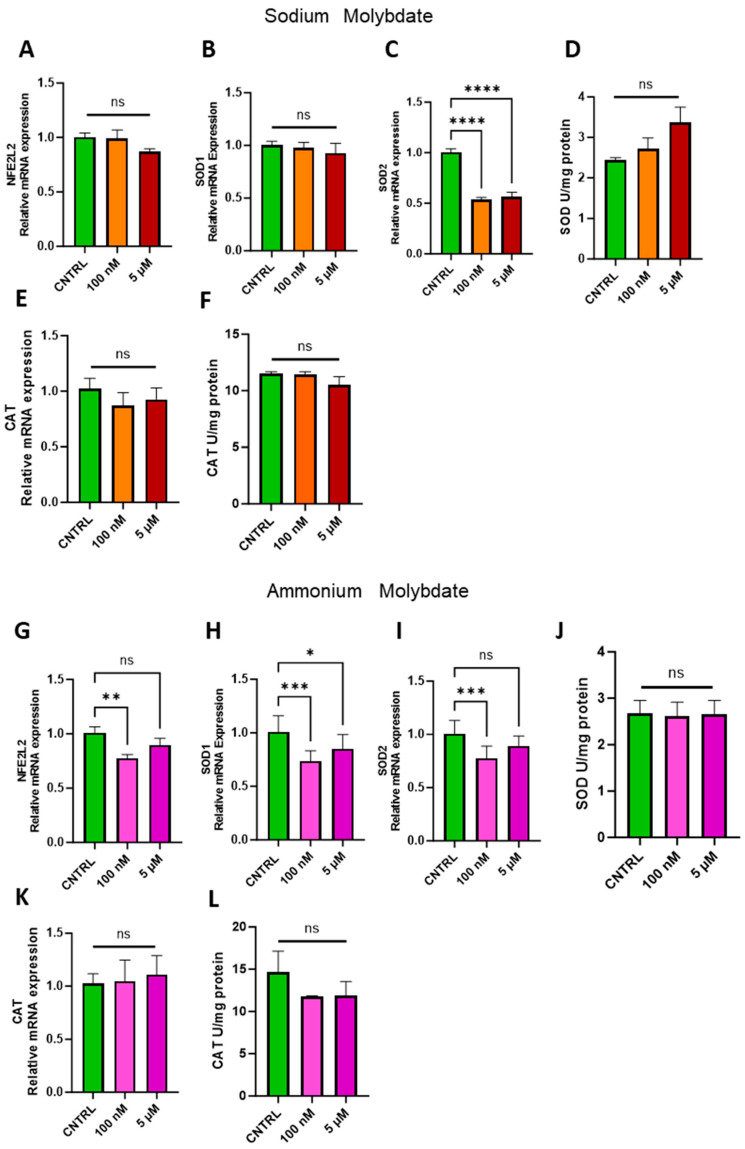
Effects of molybdate salt supplementation on antioxidant gene pathways and enzymatic activity. Messenger RNA expression of (**A**) NFE2L2, (**B**) SOD1, (**C**) SOD2, and (**E**) CAT and enzymatic activity of (**D**) total SOD and (**F**) CAT was assessed following sodium molybdate supplementation for 24 h (in orange and red). Messenger RNA expression of (**G**) NFE2L2, (**H**) SOD1, (**I**) SOD2, and (**K**) CAT and enzymatic activity of (**J**) total SOD and (**L**) CAT was assessed following ammonium molybdate supplementation for 24 h (in pink and purple). A significant *p* value less than 0.05 is signified by an asterisk (*); ** signifies *p* < 0.01, *** signifies *p* < 0.001, **** signifies *p* < 0.0001, ns indicates not significant. Data presented as mean + SEM. N = 8 separate experiments for gene expression analyses. N = 3 separate experiments for enzymatic activity analyses.

The ability of molybdenum to exert an antioxidant effect has been demonstrated in several cell lines and animal models, but it is not a universal phenomenon [1,27]. Therefore, the expression of nuclear factor erythroid 2 (NFE2)-related factor 2, coded for by *NFE2L2*, a major regulator of cellular antioxidant pathways, and notable antioxidant enzymes superoxide dismutase, *SOD1/2*, and catalase, *CAT*, were investigated in trophoblast cells following molybdenum salt supplementation (Figure 6). At both dosages of 100 nM and 5 µM sodium molybdate, no significant changes were observed in *NFE2L2* or cytosolic, copper–zinc-dependent *SOD1* (Figure 6A,B). Conversely, the expression of mitochondrial, manganese-dependent *SOD2* was significantly reduced following a 100 nM supplementation (*p* < 0.0001) and 5 µM supplementation (*p* < 0.0001) of sodium molybdate. However, total superoxide dismutase activity was not significantly modulated by molybdenum supplementation at either concentration, though the mean enzymatic activity did increase from a baseline 2.44 ± 0.074 U/mg to 3.37 ± 0.58 U/mg with 5 µM sodium molybdate supplementation (Figure 6D). The expression and activity of antioxidant enzyme catalase was also not significantly affected by either dose of sodium molybdate supplementation (Figure 6E,F), with a baseline enzymatic activity of 11.54 ± 0.19 U/mg comparable to the 5 µM supplemented catalase activity of 10.55 ± 0.98 U/mg.

Supplementation with 100 nM of ammonium molybdate resulted in the decreased expression of *NFE2L2* (*p* = 0.0089), *SOD1* (*p* = 0.0007), and *SOD2* (*p* = 0.0009) in trophoblast cells (Figure 6G–I). The expression of cytosolic *SOD1*, but not *SOD2*, was also decreased in trophoblast cells under 5 µM ammonium molybdate supplementation (Figure 6I). These changes were not reflected in total superoxide dismutase activity, which was unchanged between baseline, non-supplemented trophoblast cells at 2.68 ± 0.38 U/mg and the high ammonium molybdate-supplemented trophoblast cells at 2.66 ± 0.41 U/mg (Figure 6J). Catalase gene expression and activity were not significantly modulated by ammonium molybdate supplementation at 100 nM or 5 µM; baseline non-supplemented cells exhibited activity of 14.65 ± 3.52 U/mg, with a non-significant decrease noted in cells supplemented with 5 µM ammonium molybdate, with an average activity of 11.91 ± 2.31 U/mg (Figure 6K,L).

### 3.6. Effects of Molybdate Salts on Factors Related to Proliferation and Angiogenesis

**Figure 7:**
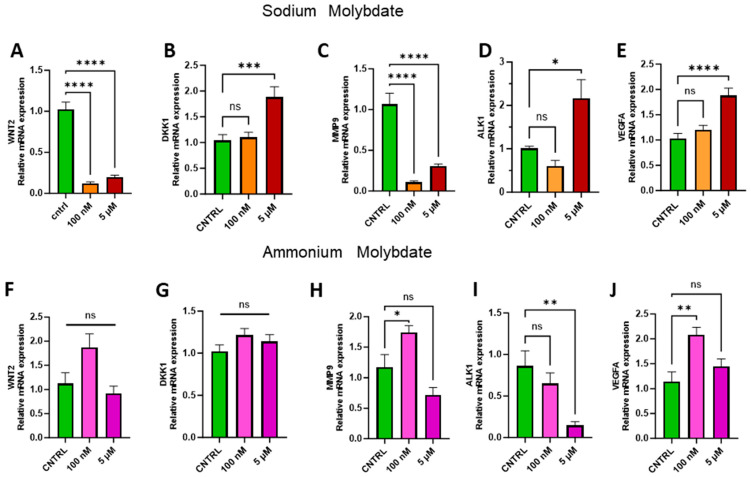
Effects of molybdate salt supplementation on proliferative and angiogenic gene pathways. Messenger RNA expression of (**A**) WNT2, (**B**) DKK1, (**C**) MMP9, (**D**) ALK1, and (**E**) VEGFA was assessed following sodium molybdate supplementation for 24 h (in orange and red). Messenger RNA expression of (**F**) WNT2, (**G**) DKK1, (**H**) MMP9, (**I**) ALK1, and (**J**) VEGFA was assessed following ammonium molybdate supplementation for 24 h (in pink and purple). A significant *p* value less than 0.05 is signified by an asterisk (*); ** signifies *p* < 0.01, *** signifies *p* < 0.001, **** signifies *p* < 0.0001, ns indicates not significant. Data presented as mean + SEM. N = 7–8 separate experiments for gene expression analyses.

Transition metals, including molybdenum, have been previously recorded to display both pro- and anti-angiogenic effects [28], with molybdenum-containing nanoparticles enhancing proliferation [29,30]. The appropriate regulation of proliferative factors such as the Wnt signalling pathway and Wnt antagonist Dickkopf-1, *DKK1*, are integral for trophoblast invasion, angiogenesis, proliferation, and placental development [31]. Therefore, the expression of *WNT2*, *DKK1*, and downstream effectors of the Wnt signalling pathway, including matrix metalloproteinase 9, *MMP9*, vascular endothelial growth factor, *VEGFA*, and activin-like kinase receptor 1, *ALK1*, were investigated to assess changes in proliferation and angiogenesis at the genetic level in trophoblast cells following molybdate salt supplementation (Figure 7).

Supplementation with 100 nM and 5 µM sodium molybdate significantly decreased the expression of *WNT2* (*p* < 0.0001, at both concentrations) (Figure 7A), while WNT-inhibiting factor *DKK1* was significantly increased following 5 µM sodium molybdate (*p* = 0.0009) supplementation only (Figure 7B). *MMP9* was modulated similarly to *WNT2* following incubation with sodium molybdate at 100 nM and 5 µM doses, with significant decreases in gene expression (*p* < 0.0001, at both concentrations) (Figure 7C). Significant upregulation of *ALK1* (*p* = 0.0135) and *VEGFA* (*p* < 0.0001) was observed with 5 µM sodium molybdate supplementation but not 100 nM supplementation (Figure 7D,E).

Ammonium molybdate supplementation did not significantly affect *WNT2* or *DKK1* expression at either concentration (Figure 7F,G). Following 100 nM ammonium molybdate supplementation, *MMP9* (*p* = 0.0302) and *VEGFA* (*p* = 0.0013) expression was significantly upregulated; this effect was not sustained at a 5 µM supplementation (Figure 7H,J). Significant downregulation of *ALK1* (*p* = 0.0024) was observed with a 5 µM supplementation of ammonium molybdate (Figure 7I).

**Figure 8:**
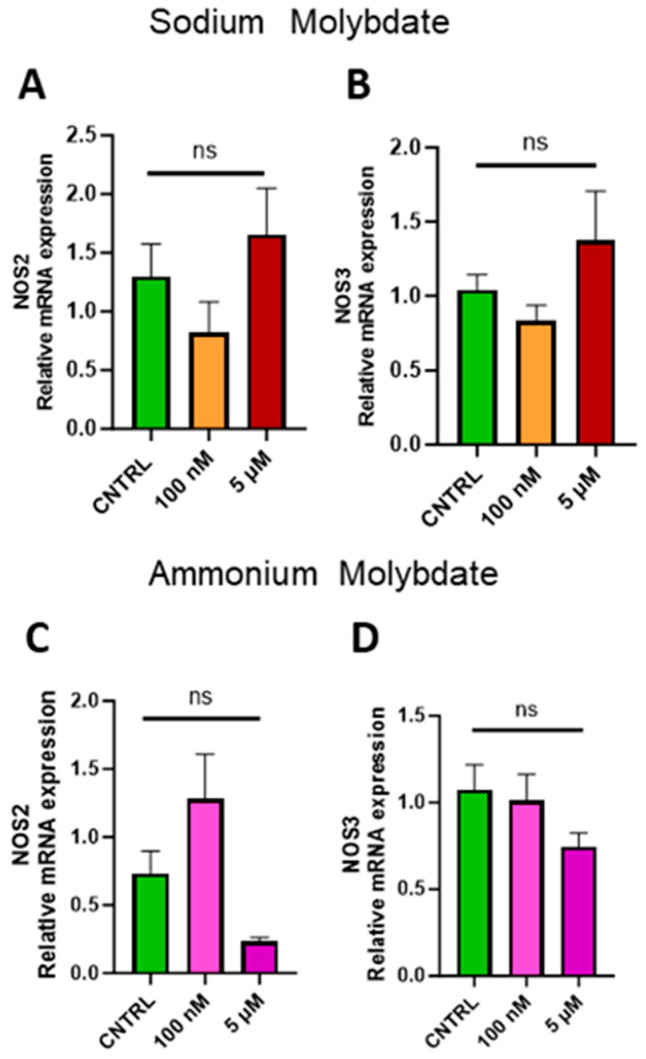
Effects of molybdate salt supplementation on nitric oxide synthesis gene expression. Messenger RNA expression of (**A**) NOS2 and (**B**) NOS3 was assessed following sodium molybdate supplementation for 24 h (in orange and red). Messenger RNA expression of (**C**) NOS2 and (**D**) NOS3 was assessed following ammonium molybdate supplementation for 24 h (in pink and purple), ns indicates not significant. Data presented as mean + SEM. N = 7–8 separate experiments for gene expression analyses.

Expression of inducible NOS (*NOS2*) and endothelial NOS (*NOS3*) were investigated to assess whether molybdate supplementation affected nitric oxide signalling at the gene level. No significant changes in gene expression were found following supplementation with sodium and ammonium molybdate at 100 nM and 5 µM concentrations (Figure 8A–D).

## 4. Discussion

This study is the first to examine the effect of the molybdate salts ammonium and sodium molybdate on HTR-8/SVneo extravillous trophoblast cells in vitro. Molybdate salts at lower physiological concentrations are not evidenced to exert any significant effects on cell growth trajectory or viability; however, this study revealed that even low physiological dosages of molybdate salts were sufficient to alter numerous gene and physiological pathways in the EVT cell line. Most notably, changes were observed in pathways relating to angiogenesis and the cellular antioxidant response, both crucial pathways for maintaining adequate placentation and foetal development. This study could therefore provide insights into the conflicting population research investigating molybdenum status and gestational health, as our data indicate that alterations in molybdate concentration or salt type can significantly alter gene and protein expression across a range of cellular mechanisms.

This study also suggests sodium molybdate may be of greater utility for the in vitro analysis of molybdate supplementation in comparison to ammonium molybdate. In accordance with previous studies on human cells, ammonium molybdate reduced cell viability and growth compared with sodium molybdate supplementation at the same supraphysiological dose (1 mM) [15]. This effect may be attributed to the increasingly acidic environment resulting from a decoupling of the ammonium ion from molybdate in solution; while not as toxic as ammonia, exposure to the ammonium ion in culture results in growth inhibition, apoptosis, intracellular acidification, and toxicity to cells at sufficiently high concentrations [32,33]. We can infer that the nanomolar, micromolar, and low millimolar dosages of molybdenum present in the study, in the form of sodium molybdate salt, are tolerated well by trophoblast cells based on viability and cell growth data.

The difference between salts is furthermore notable at the physiological supplementation level; gene expression changes are not comparable between the two salts for any of the pathways analysed, with the exception of xanthine oxidase activity. We postulate that these differences could arise from a combination of a cellular response to the increasingly acidic cell culture medium and the reduced utilisation of molybdate by the cell due to the decrease in pH, which decreases molybdate solubility and increases the interaction with metal oxides, reducing bioavailability [34]. In plants, molybdate was found to be bioavailable in soil at a neutral to slightly alkaline pH [35]. Although to date, there have been no studies concerning molybdate absorption in mammalian cells in a pH-dependent manner, considering the tight regulation of acid-base chemistry by mammalian cells, even small perturbations in pH may affect molybdate acquisition. This may be reflected in the significantly increased expression of molybdenum cofactor genes *MOCS1* and *MOCS2* following ammonium molybdate supplementation at the higher salt dose of 5 μM (Figure 5D), which we postulate as a cellular response to decreased available molybdate in cell culture.

As key players in cellular function and development, molybdoenzymes xanthine oxidase, aldehyde oxidase, and sulphite oxidase were studied at the gene, protein, and activity levels. Three of the currently known molybdoenzymes, *MARC1*, *MARC2*, and *AOX1*, were not significantly increased at the gene level following molybdate salt exposure in trophoblast cells. Further, expression of *XOR* was unaffected by supplementation with sodium molybdate and *SUOX* unaffected by supplementation with ammonium molybdate. An amount of 100 nM of sodium molybdate increased protein levels of xanthine dehydrogenase, but supplementation with ammonium molybdate decreased sulphite oxidase and xanthine oxidase protein levels. The dearth of in vitro literature examining mammalian cell exposure to molybdate does not allow for much comparison; however, there is existing evidence that molybdenum supplementation can increase molybdoenzyme expression and activity [35]. It is intuitive to expect this as a consistent phenomenon, considering other micronutrient supplementation studies of elements such as selenium increasing enzymatic expression and activity following supplementation [36,37]. For molybdenum however, this effect has not been observed consistently across the literature [38]. A reduction in protein levels and enzymatic activity may be induced by a heightened molybdenum availability that exceeds cellular storage capacity.

Molybdenum is furthermore demonstrated to exert pleiotropic effects in cells in conjunction with the molybdoenzyme function, notably in augmenting the antioxidant response [39]. High molybdenum exposure increased the expression and activity of antioxidant enzymes such as superoxide dismutase and catalase in a range of cell and animal models [1,18,39]. Crucially, this effect was observed at high dosages, and also investigated specifically after an induced cellular insult. The antioxidant response following low-level exposure in complete cell media independent of induced oxidative stress has not been investigated. Our study reveals that sodium molybdate supplementation induced a reduction in mitochondrial enzyme *SOD2* at nanomolar exposure, which may relate to a reduced requirement for the enzyme, as reflected in the non-significant increase in enzyme activity at the highest molybdate supplementation. However, supplementation with a nanomolar dose of ammonium molybdate significantly reduced the expression of several antioxidant genes, including antioxidant transcriptional regulator *NFE2L2* (Nrf2) and downstream targets *SOD1/2* and *CAT*, indicative of perturbations in the global antioxidant response following supplementation. While no changes were observed in viability and proliferative parameters in this study, these data suggest the antioxidant capacity of trophoblast cells may be affected by exposure to ammonium molybdate in vitro.

Molybdenum salt exposure is also associated with both pro- and anti-angiogenic effects [28], which vary with salt type. As a highly vascularised organ, the placenta is a rich source of both pro- and anti-angiogenic factors, which are essential for the maintenance of blood flow and nutrient exchange during gestation [40]. Sodium molybdate supplementation in the present study decreased the expression of angiogenic, proliferative and remodelling factors *WNT2* and matrix metalloprotease 9 and increased the expression of *DKK1*, a primary Wnt antagonist, and *ALK1*, an anti-angiogenic signalling molecule. Taken together, it is clear that sodium molybdate significantly inhibits angiogenesis signalling at the gene expression level in vitro. Whether this response would be replicated in vivo requires further investigation but raises questions as to the utility of molybdenum supplementation, especially during the dynamic process of angiogenesis required for healthy placentation.

Unexpectedly, angiogenic factor *VEGFA* was upregulated with exposure to sodium molybdate. The transcriptional upregulation of *VEGF* is mediated by the Wnt signalling pathway [41] but also independently of it through IRE1α, PERK, and ATF6α, master regulators of the unfolded protein response (UPR) pathway, which activates following endoplasmic reticulum (ER) stress and is integral to placental development and the placental response to adverse events [42]. Alterations to the UPR pathway have previously been reported following molybdenum exposure (Mo^6+^) at 0.25 mg/L dosages in a male mouse model, as a result of ER stress [43]. In the present study, whether this upregulation of VEGFA in EVT cells is due to an increase in endoplasmic reticulum stress as a result of molybdenum supplementation, or an unelucidated response to molybdate in general, is currently difficult to discern. Ammonium molybdate supplementation inversely upregulated the expression of *MMP9* and decreased the expression of *ALK1*, while *WNT2* and *DKK1* remained unaffected, indicative of a pro-angiogenic phenotype at the gene level and further suggesting that cellular response to the molybdate ion is modulated by associated ions in salt solution and possible pH alterations. Further research is necessary to explore the full effects of molybdate supplementation on placental proliferation and invasion; the dysregulated expression of *WNT* and *DKK1* during pregnancy is associated with shallow placentation and pre-eclampsia (reduction) but also with gestational trophoblastic diseases such as molar pregnancy (excess) [39]. Trophoblast implantation and placental vasculogenesis and angiogenesis are furthermore modulated by the endogenous vasodilator nitric oxide (NO), which can interact with angiogenic growth factors such as *VEGF* [44]. Molybdoenzymes have been evidenced to act as nitrite reductases, producing nitric oxide in a complementary fashion to traditional NO synthesis via the nitric oxide synthase, *NOS*, family of enzymes [45]. No alterations in the expression of nitric oxide synthesis were observed in the present study.

Ammonium molybdate has previously been administered as a molybdenum supplement following deficiency, with no adverse effects reported in patients or in agricultural animals, and both salts have similar bioavailability values from a single study performed in lambs [46,47]; however, it is notable that gestation, as a prolonged period of high metabolic activity, can lead to acidosis or alkanosis states. As a result of foetal hypoxia and reduced placentation, an excess of organic acids such as lactic acid can deplete maternal and foetal buffer systems, resulting in acidosis, which is associated with poor Apgar scores and gestational diseases [48]. Nausea and vomiting during pregnancy can also affect maternal–foetal acid-base chemistry and deplete molybdenum reserves, resulting in unexpected molecular alterations.

### Study Limitations

The authors acknowledge that this study was performed using HTR-8/SVneo; an immortalised cell line of first-trimester extravillous trophoblast cells cannot accurately reflect all placental cellular functions throughout gestation [49]. However, the examination of HTR-8 cells in this context remains relevant, as extravillous trophoblast cells are key players in decidual invasion and placental development. Additionally, the existing literature examining molybdenum content and its relation to gestational health complications, such as metabolic dysfunction, have speculated that molybdenum may exert a protective effect in the first trimester [1].

The present study utilised molybdenum salts as previously described in a seminal study examining the genotoxicity of molybdenum salts in murine and human cells [17]. Although beyond the scope of an initial characterisation in human trophoblast cells, assessing molybdenum content in cell media components such as foetal bovine serum, which is known to contain variable molybdenum concentrations, via ICP-MS, should be employed in future investigations [50]. Now a baseline function has been established, chelators to completely deprive cell lines of any potential molybdenum content could provide further insight into the mechanisms of molybdate in future. However, regardless of the trace content of molybdenum in culture media, we are substantially increasing the concentration and availability of the element in our study, and by a consistent value.

Subsequent studies investigating molybdenum salt supplementation would benefit from additional controls of ammonium and sodium at an equivalent molarity. To fully elucidate whether the effects observed are a result of molybdenum supplementation or could be phenocopied with sodium or ammonium supplementation alone, or a synergistic effect of both molecules, is a potential avenue of research. This is especially pertinent to clarify, considering that ammonium chloride supplementation was previously established to inhibit growth in a range of cell types [51,52]. Significant changes in intracellular pH as a result of ammonium exposure are likewise well established and formed the basis for our hypotheses regarding molybdenum bioavailability in this study; however, additional analyses of media pH at regular intervals during salt supplementation would be beneficial in expanding our understanding of molybdate metabolism in mammalian cells under conditions of acidosis and alkalosis. This could be combined with analytical methods such as ICP-MS to quantify intracellular molybdate stores following varied molybdenum salt supplementation.

## 5. Conclusions

This study was the first to utilise molybdenum salts at levels that do not induce overt toxicity in a trophoblast cell model, and it showcased the effects of molybdenum salts on gene expression, protein expression, and enzymatic activity in an extravillous trophoblast cell line. The HTR-8/SVneo cell line is known to correlate with in vivo trophoblast behaviour and respond similarly to chemical stimuli [53]. The differences in ammonium and sodium molybdate salt behaviour may have potential in the research of molybdenum dynamics in vivo; if molybdate signalling in mammalian cells is influenced by even small perturbations in pH or the presence of other ions, it could be a potential avenue of research into the often contradictory correlations of molybdenum concentrations with disease states in population studies [1].

This foundational study therefore sheds light on and invites further investigation into molybdenum exposure during pregnancy. Molybdenum is an essential micronutrient, and at sufficiently high concentrations following oxidative stress insult, it exhibits antioxidant potential; however, at low exposure levels and in a non-oxidatively stressed environment, antioxidant activity was not significantly altered, and reductions in angiogenesis-related gene pathways were noted. The cellular response to molybdenum supplementation is likely influenced by oxidative stress levels, which further raises questions regarding the benefit of molybdenum supplementation in the general pregnant population versus targeted supplementation for complicated pregnancies. Gene expression changes were not altered in a dose-dependent manner, and frequently, lower concentrations of molybdate salt exerted greater effects. Alongside this observed non-monotonic characteristic, molybdenum is also evidenced to have a very narrow efficacy range—fundamental research could aid in untangling the conflicting findings of molybdenum exposure during pregnancy at the human population level. Our study, in conjunction with the existing data, provides evidence for the pleiotropic effects of molybdenum at a range of concentrations and under varied conditions. Further study is required to examine these complex environmental interactions with placental physiology. In order to contextualise these findings further, a cell stress model could be implemented to examine the effect of molybdenum supplementation under environments of heightened oxidative stress, which may further reveal beneficial or adverse effects during pregnancy.

## Figures and Tables

**Table 1:** PCR primer sequences used for examination of genes relating to molybdenum cofactor synthesis, molybdoenzymes, antioxidant response, proliferation, and angiogenesis, and housekeeping genes for normalisation of PCR results.

	Gene Name	Primer Sequence	Protein Name
MoCo Synthesis	MOCS1	F 5′-CAGGCATGTTCAGTATTTCC-3′	Molybdenum Cofactor 1
		R 5′-CATCCTTTCCATAAAGTGGC-3′	
	MOCS2	F 5′-GGTGCAATATCCCTATTTGTAG-3′	Molybdenum Cofactor 2
		R 5′-AACACTGCTATGTGTTTGAC-3′	
	GPHN	F 5′-ACAGGTAATCAAATGAGCAG-3′	Gephyrin
		R 5′-TATGTGGACATGCATCAAAG-3′	
Molybdoenzymes	MARC1	F 5′-CAGATTGCTTACTCAGACAC-3′	Mitochondrial Amidoxime Reducing Component 1
		R 5′-GCTTTAACTTTCTTCTCTAGCC-3′	
	MARC2	F 5′-CTGGGATGAACTCCTAATTG-3′	Mitochondrial Amidoxime Reducing Component 2
		R 5′-TTTCCTGTCTATGACTCCAG-3′	
	AOX1	F 5′-GAGAATGATGTGGTTTCCC-3′	Aldehyde Oxidase 1
		R 5′-TTAAATTTCACTTCAGGCCC -3′	
	XOR	F 5′-CTACAGCTTTGAGACTAACTC-3′	Xanthine Oxidoreductase
		R 5′-TCTTATGATCTCCTGTTAGGC-3′	
	SUOX	F 5′-GACTCAAGTCAATCCCCTC-3′	Sulfite Oxidase
		R 5′-CATGACTCTCCATCCCTG-3′	
Antioxidant	NFE2L2	F 5′-CGTTTGTAGATGACAATGAGG-3′	Nuclear factor erythroid-derived 2-like 2
		R 5′-AGAAGT7CAGGTGACTGAG	
	SOD1	F 5′-GAGCAGAAGGAAAGTAATGG-3′	Superoxide Dismutase 1
		R 5′-GATTAAAGTGAGGACCTGC-3′	
	SOD2	F 5′-ATCATACCCTAATGATCCCAG-3′	Superoxide Dismutase 2
		R 5′-AGGACCTTATAGGGTTTTCAG-3′	
	CAT	F 5′-CTCTTCTGGACAACTACAATG-3′	Catalase
		R 5′-AGGAGAATCTTCATCCAGTG-3′	
Proliferation and Angiogenesis	WNT2	F 5′-TTAATATGAACGCCCCTCTC-3′	Wingless-type MMTV integration site family, member 2
		R 5′-TACCACCATGAAGAGTTGAC-3′	
	DKK1	F 5′-GGACAAGAAGGTTCTGTTTG-3′	Dickkopf WNT signalling pathway inhibitor 1
		R 5′-CTTCTTTCAGGACAGGTTTAC-3′	
	MMP9	F 5′-AAGGATGGGAAGTACTGG-3′	Matrix metallopeptidase 9
		R 5′-GCCCAGAGAAGAAGAAAAG-3′	
	VEGFA	F 5′-AATGTGAATGCAGACCAAAG-3′	Vascular endothelial growth factor A
		R 5′-GACTTATACCGGGATTTCTTG-3′	
	NOS2	F 5′-AGCTCAACAACAAATTCAGG-3′	Nitric oxide synthase 2
		R 5′-ATCAATGTCATGAGCAAAGG-3′	
	NOS3	F 5′-CAACCCCAAGACCTACG-3′	Nitric oxide synthase 3
		R 5′-CGCAGACAAACATGTGG-3′	
Housekeepers	GAPDH	F 5′-TCGGAGTCAACGGATTTG-3′	Glyceraldehyde-3-phosphate deydrogenase
		R 5′-CAACAATATCCACTTTACCAGAG	
	ACTB	F 5′-GACGACATGGAGAAAATCTG-3′	Beta-actin
		R 5′-ATGATCTGGGTCATCTTCTC-3′	

## Data Availability

The original contributions presented in this study are included in the article. Further inquiries can be directed to the corresponding author.

## Abbreviations

The following abbreviations are used in this manuscript:

ACTB	Beta-actin
AOX	Aldehyde oxidase
ATCC	American Type Culture Collection
AUC	Area under curve
CAT	Catalase
DKK1	Dickkopf WNT signalling pathway inhibitor 1
DMSO	Dimethyl sulfoxide
EDTA	Ethylenediamine tetraacetic acid
EVT	Extravillous trophoblast
FBS	Foetal bovine serum
FGR	Foetal growth restriction
GAPDH	Glyceraldehyde-3-phosphate dehydrogenase
GEPH	Gephyrin
kDA	Kilodalton
MARC1/2	Mitochondrial amidoxime reducing component 1/2
MMP9	Matrix metallopeptidase 9
Mo	Molybdenum
Moco	Molybdenum cofactor
MOCS1/2	Molybdenum cofactor synthesis 1/2
MPT	Molybdopterin
MTT	3-(4,5-Diemthylthiazol-2-Yl)-2,5-Diphenylterazolium Bromide
NFE2L2	Nuclear factor erythroid-derived 2-like 2
NOS2	Nitric oxide synthase 2
NOS3	Nitric oxide synthase 3
PBS	Phosphate-buffered saline
qRT-PCR	Quantitative real-time polymerase chain reaction
RPMI	Roswell Park Memorial Institute (media)
SOD1	Superoxide dismutase 1
SOD2	Superoxide dismutase 2
SUOX	Sulphite oxidase
TBST	Tris-buffered saline with 0.1% Tween^®^ 20 detergent
U	Unit
UPR	Unfolded protein response
V	Volt
VEGFA	Vascular endothelial growth factor A
WNT2	Wingless-type MMTV integration site family, member 2
XDH	Xanthine dehydrogenase
XO	Xanthine oxidase
XOR	Xanthine oxidoreductase
